# Introduction of a human- and keyboard-friendly N-glycan nomenclature

**DOI:** 10.3762/bjoc.20.53

**Published:** 2024-03-15

**Authors:** Friedrich Altmann, Johannes Helm, Martin Pabst, Johannes Stadlmann

**Affiliations:** 1 Department of Chemistry, BOKU University, Vienna, Austria; 2 Department of Biotechnology, Delft University of Technology, Delft, The Netherlands

**Keywords:** N-glycans, nomenclature, structural features

## Abstract

In the beginning was the word. But there were no words for N-glycans, at least, no simple words. Next to chemical formulas, the IUPAC code can be regarded as the best, most reliable and yet immediately comprehensible annotation of oligosaccharide structures of any type from any source. When it comes to N-glycans, the venerable IUPAC code has, however, been widely supplanted by highly simplified terms for N-glycans that count the number of antennae or certain components such as galactoses, sialic acids and fucoses and give only limited room for exact structure description. The highly illustrative – and fortunately now standardized – cartoon depictions gained much ground during the last years. By their very nature, cartoons can neither be written nor spoken. The underlying machine codes (e.g., GlycoCT, WURCS) are definitely not intended for direct use in human communication. So, one might feel the need for a simple, yet intelligible and precise system for alphanumeric descriptions of the hundreds and thousands of N-glycan structures. Here, we present a system that describes N-glycans by defining their terminal elements. To minimize redundancy and length of terms, the common elements of N-glycans are taken as granted. The preset reading order facilitates definition of positional isomers. The combination with elements of the condensed IUPAC code allows to describe even rather complex structural elements. Thus, this “proglycan” coding could be the missing link between drawn structures and software-oriented representations of N-glycan structures. On top, it may greatly facilitate keyboard-based mining for glycan substructures in glycan repositories.

## Introduction

**Figure 1:**
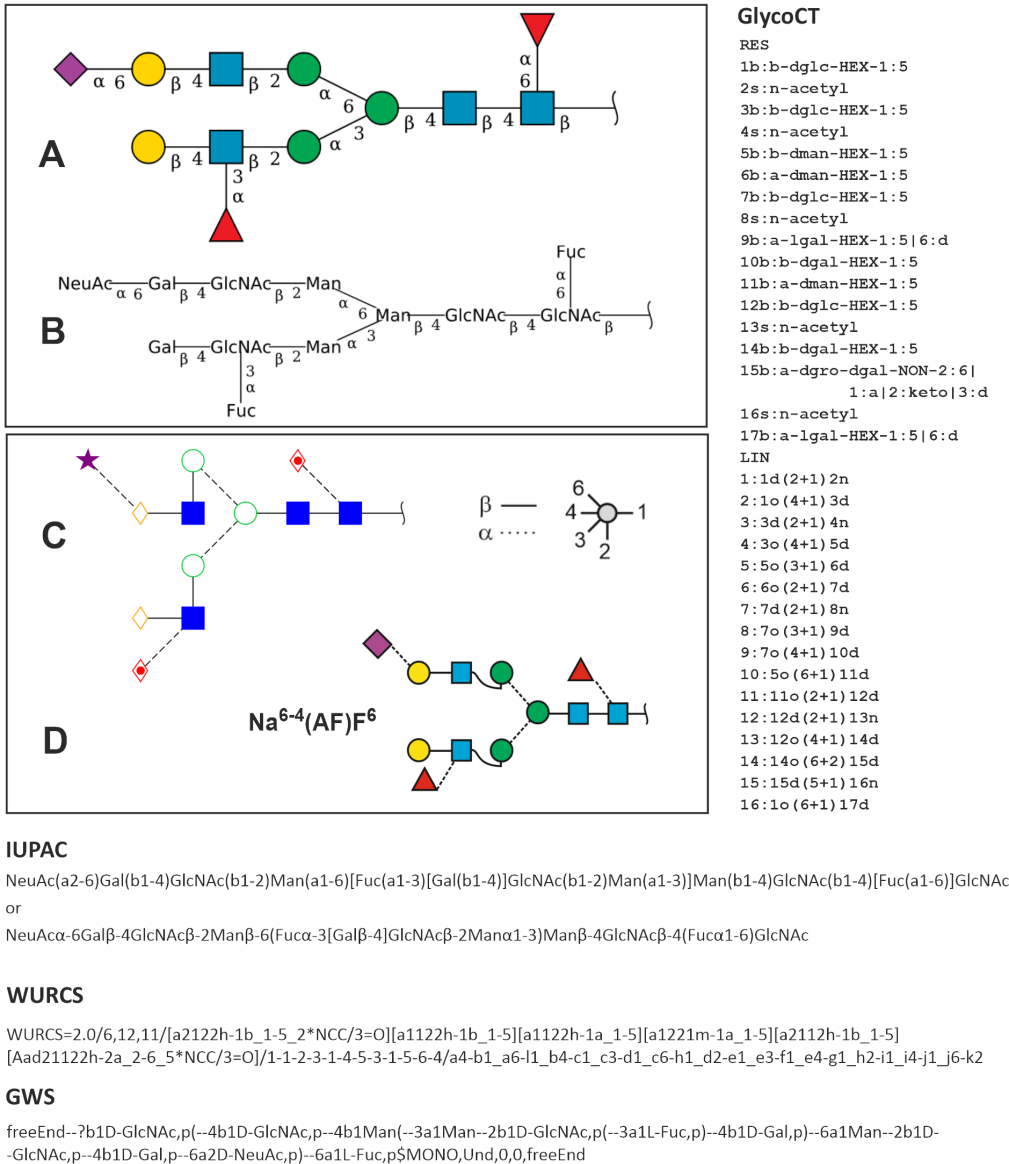
Descriptions and depictions of a particular complex-type N-glycan structure found in human leukocytes [2]. A: SNFG depiction; B: IUPAC depictions; C: Oxford style; D: smart SNFG. Except for 1D, cartoons and codes were adapted from [3] (© GlyConnect v1.2.0, distributed under the terms of the Creative Commons Attribution 4.0 International License, https://creativecommons.org/licenses/by/4.0). Cross-references in other data repositories are: GlyTouCan [4], GlyGen [5], ChEBI [6].

Virtually any article on protein glycosylation starts with imposing assurances about the biological significance of the various structures. This contrasts with the strange awkwardness when structures of interest, e.g., a possible biomarker, shall be named. Uncompromising carbohydrate chemists will use chemical formulas drawn with ChemDraw, ChemSketch or alike. While this generates rather bulky pictures, chemists rely on the possibility to specify the chemical peculiarities deliberately introduced by their sorcery. Biochemists and medical chemists, however, rather focus on the native glycan structure as grafted on the protein substrate by the biosynthetic machinery. Thus, biochemists could do with the much simpler IUPAC code [1] (Figure 1), which has the invaluable advantage of being an alphanumeric code and which nowadays mostly dispenses use of Greek letters for annotating anomericity.

Look – as an example – at entry G75903TQ in the relevant database GlyTouCan (glytoucan.org) [7] for the structure that we will herein baptize **Na^6-4^(AF)F^6^** and ask yourself, how one could label an Eppendorf vial with the most condensed approved version of the IUPAC code (Figure 1). The graphical depictions are far more illustrative. On top, the “Symbol Nomenclature for Glycans” (SNFG) format, which was pioneered by the Consortium of Functional Glycomics (CFG), with its logical and appealing monosaccharide symbols has fortunately now become standard. Cartoons like this can be generated by GlycanBuilder [8–9], Glyconnect [10], GlyTouCan [7], GlycoWorkbench [9], SugarSketcher [11–12] or GlycoDraw [13] or by a universal graphics program such as, e.g., CorelDraw. The latter allows to merge the SNFG format with Oxford style thereby offering the particular advantage of encoding linkage position and anomericity in the cartoon itself obviating the need for letters, which require a minimal font size to remain readable. The resulting “smart”-SNFG thus transports structural information on a smaller space while essentially retaining the familiar SNFG appearance (Figure 1D). This is, however, just a shy suggestion with no ties to the actual topic of this treatise, even though others have already applied the same logic [14]. The cumbersome struggle for glycan depictions and related software has lately been portrayed in a quite enjoyable review [15].

A number of computer-readable formats has been developed of which GlycoCT [16] and WURCS [17] are currently the options of choice. None of these software friendly options is even remotely suitable for labeling of spectra or vials. Therefore, scientists in the huge field of N-glycan research have soon begun to use acronyms. One early adopted system was developed for the antibody field, where basically the number of galactose residues was annotated. In the most fully developed format, the presence of fucose and sialic acid is deliberately mentioned [18] (see also Table 1).

A more versatile set of annotation systems was built around the number of antennae. The term 3A2S denotes a triantennary N-glycan with two sialic acid residues. Obviously, this lean annotation does at first not specify linkage types and branch allocation. Therefore, add-ons were developed to allow a more precise description [19–24]. A structure probably representing the one shown in Figure 1 was described as FcA2G2FS [24]. While concise and informative, several details still remain undefined and we cannot see how the basic architecture of this system could be further developed.

To summarize, a human-, vial- and keyboard-friendly abbreviation system that nevertheless transports unequivocal structural information is still missing. The following chapters will pursue the question if the “proglycan” abbreviation system could be a useful tool, when N-glycan structures shall be talked about, written on vials or in texts or put in tables. This system has proven useful for communication with partners for already many years. The terms MMXF^3^ and MUXF^3^ (with or without the superscript number) enjoy widespread use in the allergy diagnosis community [25].

Our recent working with 40 isomeric N-glycans all composed of 5 hexoses, 4 HexNAcs and 1 fucose [26–27] reinforced our convictions that a simple and logical naming system is required and that the “proglycan” system could meet the demands. In the following chapters, aspects of how to name structures with different types of fucosylation, sialylation or antennary branching, bisecting GlcNAc, GalNAc, oligomannosidic structures and more will be discussed.

## Discussion

### The beginnings and the basics

In the 90ties during a guest visit in our lab, Prof. Harry Schachter from Toronto University taught us the term GnGn for the acceptor substrate of fucosyl transferases [28–29]. This “*reductio ad essentialia*” proved very helpful in our work with glycosyltransferases, e.g., insect hexosaminidase [30] and plant fucosyltransferase [31], which dealt with terminal modifications of always the same core. Years later, colleagues from in- and outside my institution engaged in “humanization” of plant N-glycans, which at first entailed removal of the plant-typical residues α1,3-fucose and xylose [32–33]. Later, the glyco-engineering of plants with the introduction of human features such as β1,4-galactose, branched and bisected glycans and sialylation required numerous experiments and a handy annotation of dozens of unusual structures for vials and quick notes on paper as well as papers [34–37]. This made us increasingly confident of the underlying principle of the “proglycan” nomenclature, an acronym derived from our then *nom de guerre* “protein-glycosylation analysis” group. By the way, glycan analysis also funneled in activities in the area of allergy diagnosis, where the term MUXF^3^ enjoys widespread use [38–40]. Finally, our work on the isomer-specific analysis of glycoproteins solidified our faith in a rather universal applicability of this system [26–27].

**Figure 2:**
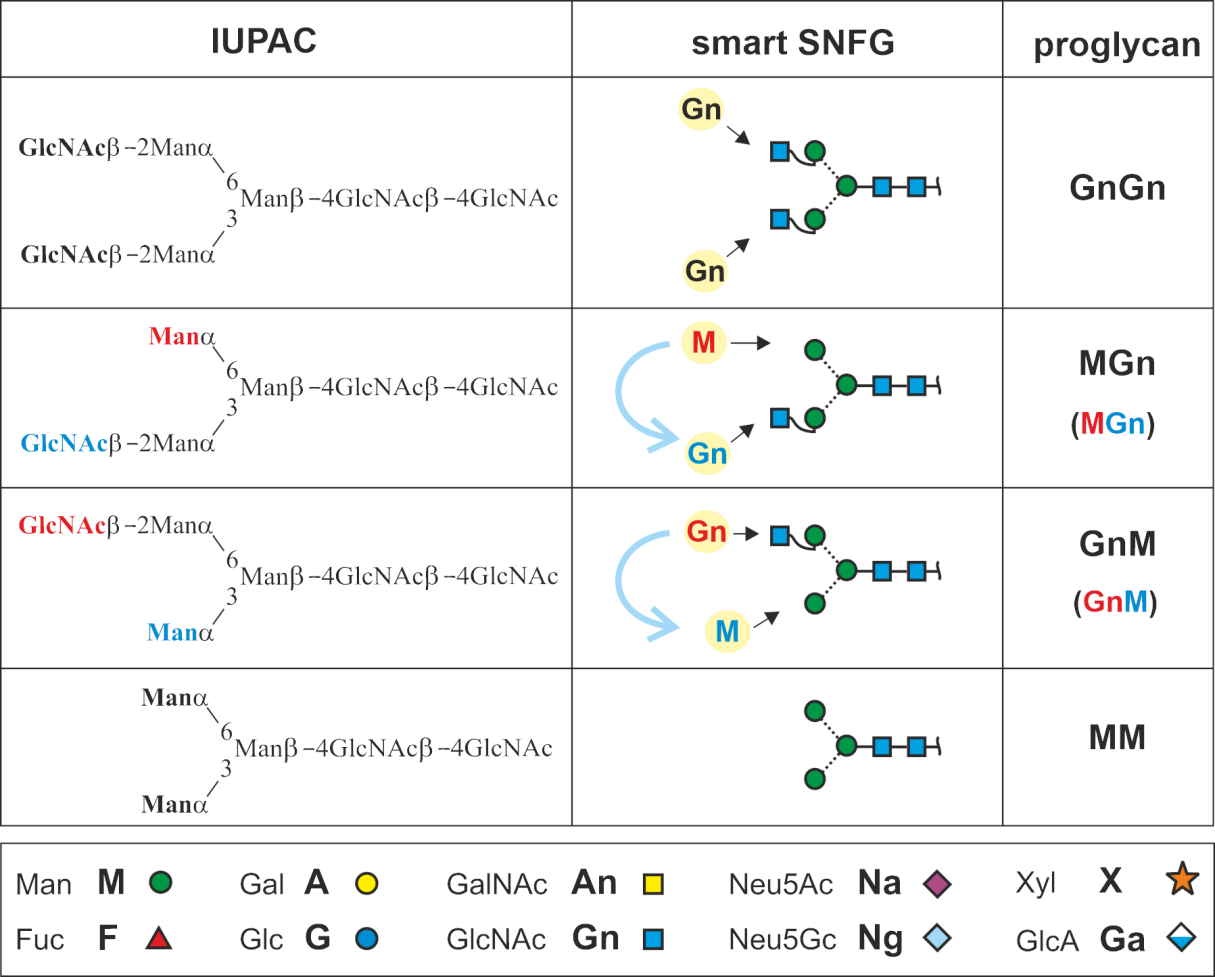
The initial idea of describing an N-glycan just by its terminal residues. Reading the termini from top to bottom = counter-clockwise introduces arm specificity. The box at the bottom connects abbreviated monosaccharide names with proglycan shorthand names and SNFG symbols.

But now, back to the meaning of “GnGn”. Gn stands for GlcNAc and the two Gn-s symbolize the two terminal residues of a biantennary N-glycan (Figure 2). The GlcNAcs are attached to the common core pentasaccharide. No further definitions are required as the residues preceding the GlcNAcs are unambiguously determined by the biosynthetic pathway of N-glycans. Action of hexosaminidases would generate two isomers with just one GlcNAc and a terminal mannose residue. By defining the reading direction, we can easily distinguish these two isomers (Figure 2).

### Galactosylation (including the alpha-Gal epitope) and sialylation

**Figure 3:**
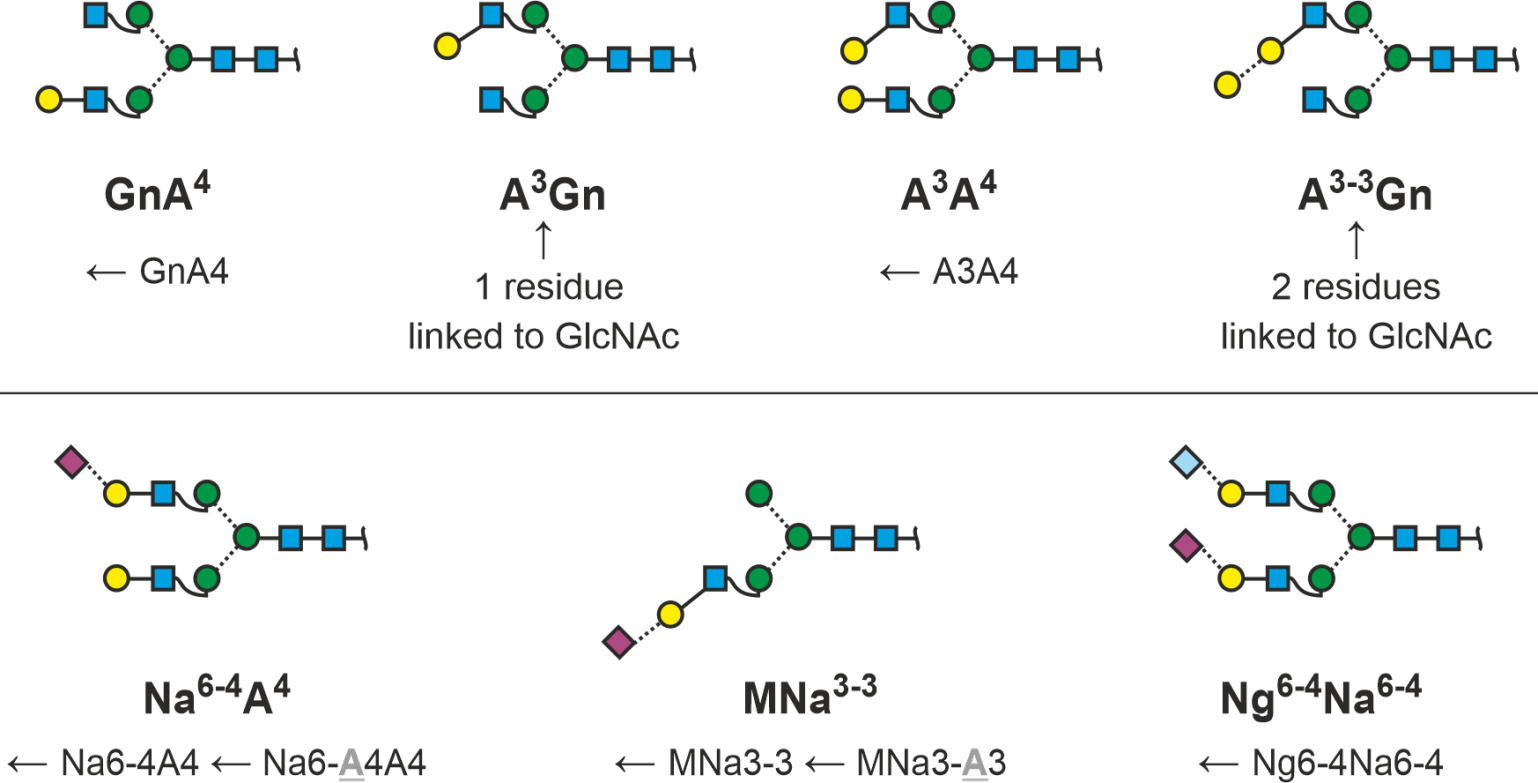
Annotation of antennae with galactose and sialic residues with consideration of linkage options. Note that the use of superscript format for linkages is recommended style but optional as it does not convey information.

Often, the antennae are elongated by galactose whose one-letter code [41] is A. Added to the acceptor structure GnGn (Figure 2) this leads to three possible products if we only consider β1,4-linkages. Rarely, however, the linkage is β1,3. To account for this ambiguity, we use a number defining to define the point in question, in this case 3 or 4 (Figure 3). Writing these numbers as superscripts provides a more comprehensible structure to the term, but it is in no way crucial for the information content.

We now know the three basic terminal elements M, Gn and A (either A^3^ or A^4^) of an antenna. It is clear by definition that “Gn” and “A^4^” annotate the sequences GlcNAc(β1-2)Manα1- and Gal(β1-4)GlcNAc(β1-2)Man(α1-, respectively. Whenever we further elongate the sequence, we need to consider the Gal(β1-…) residue. The first example for this necessity is the Galα1,3-Galβ1- element. We might resort to something like Aα1-3A^4^ or Aα1-3A^3^. The suggested annotation, however, is A^3-4^ or A^3-3^ [26], whereby the two figures connected by a hyphen point at a disaccharide unit. As alpha-Gal is only known as linked to a beta-Gal residue, this lean annotation is unambiguous unless – very unexpectedly – a novel structural element turns up that makes this spelling invalid.

Sialic acids – usually – are likewise linked to the β-galactose. Sialic acids may occur in the 3- or in the 6-position of this Gal residues. Following the example of alpha-Gal residues, we can write a Neu5Ac(α2-6)Gal(β1-4)GlcNAc(β1-2) antenna as Na^6-4^ while retaining the complete structural information (Figure 3). Rarely, a sialic acid residue is linked directly to GlcNAc as in bovine fetuin [42]. As this element was found on triantennary N-glycans only, we will deal with it in the relevant chapter below.

### Core-fucosylation, bisecting GlcNAc and xylose

**Figure 4:**
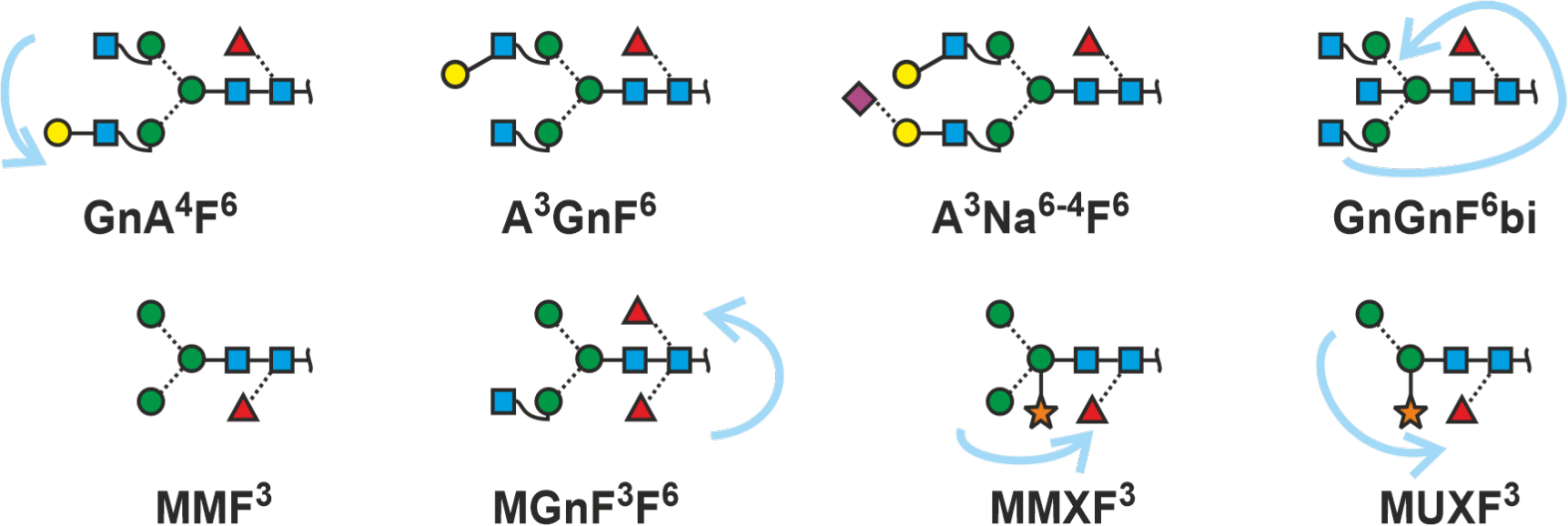
Core substituents are listed in counter-clockwise order.

The core fucose constitutes a third terminal residue and hence we introduce a third structure term, “F” for fucose. We could simply write, e.g., A^4^A^4^F. In mammals, the core fucose is strictly always in α1,6. If you only work with mammalian samples you may content yourself with this simplification. However, as insect cells and plants are of relevance as biotechnological expression systems for pharmaceutical glycoproteins, we must consider that here fucose may also be found in the α1,3-position. Therefore, superscripts should be used to define the type of core fucose (Figure 4).

A structural element frequently occurring in mammalian glycoproteins, e.g., in immunoglobulins is the bisecting GlcNAc. This shall be added at the end of the abbreviation string by two lower case letters “bi”. In plants and some non-vertebrate animals [43–44], the β-mannosyl residues may instead be decorated with a xylose residue, for which the letter X is added after the term for the 3-arm (Figure 4).

### Fucose on antennae

**Figure 5:**
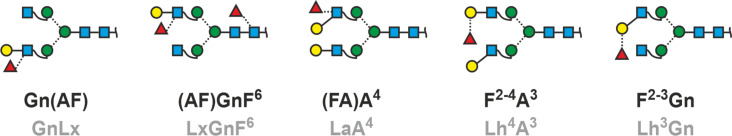
Terminal fucosylation depicted with standard abbreviations and with ”macro-terms”.

Lewis fucoses introduce branching of the antenna. IUPAC nomenclature uses square brackets to identify a branch. We do something similar. However, the two residues, or chains, that are linked to the root of the branch are both put in round brackets. The substitution points are defined by superscripts. So, LeX fucosylation of an A^4^ antenna transforms this term to (A^4^F^3^). A LeA structure would be (F^4^A^3^) because by default we read the structure counter-clockwise. However, we want to be as elegant and concise as possible. Are we losing any information when omitting the superscripts? Unless novel structural peculiarities of N-glycans emerge, the answer is no. Therefore, we simply write (AF) and (FA) (Figure 5).

Another difficulty is posed by the blood group H α1,2-fucose, which is linked to galactose, which in turn can be linked β1,3- or β1,4 to GlcNAc. So just putting “F” as the terminal sugar would leave uncertainty. Therefore – using linear code [41] – we write F^2^-A^4^. We – again – can save one character by omitting the A to arrive at: F^2-4^ or F^2-3^ (Figure 5). Why not just F^4^ or F^3^? Because we must not ignore the galactose. “F^4”^ would be a Fuc(α1-4)GlcNAc substructure, which does not exist.

**Figure 6:**
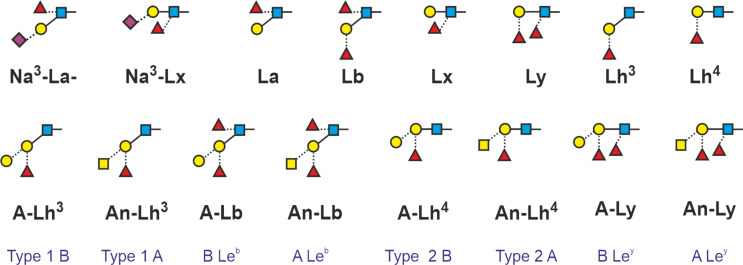
Suggested annotations of ABO and Lewis blood-group antigens using the “macro-terms” La, Lb, Lx, Ly and Lh. Traditional designations are shown in blue [45].

In a rush of boldness, we felt tempted to introduce “macro-terms” for relevant structural elements. The LeX and LeA determinants (AF) and (FA) could then be pointed up as Lx and La (Figure 5, grey terms). In fact, this logic can be the key for depicting the entire range of Lewis and blood-group antigens as suggested in Figure 6.

### Multi-antennary glycans

**Figure 7:**
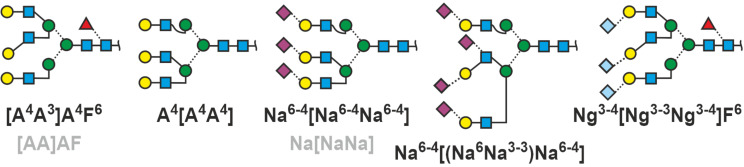
Multi-antennary N-glycans. In addition to regular proglycan abbreviation, intended ambiguity style (see chapter below) is shown in grey for selected structures. The colored codes are just for illustration.

A branch resulting in a triantennary structure can occur on either arm of an N-glycan. The two antennae ascending from the same mannose are set in square brackets (Figure 7). The square exclusively and immediately tells us that this branch is further branched. By that the two basic types of triantennary glycans are readily told apart.

The proglycan nomenclature reaches its limits here because the terms become lengthy and difficult to read, but then, what is the alternative? Even the strange tetrasialylated triantennary glycan in bovine fetuin can be depicted. Note that the term [(Na^3-3^Na^6^)Na^6-4^] contains – inside the square brackets – round brackets signifying additional branching. As no other branching point is given, the root residue is GlcNAc.

### LacNAc repeats

**Figure 8:**
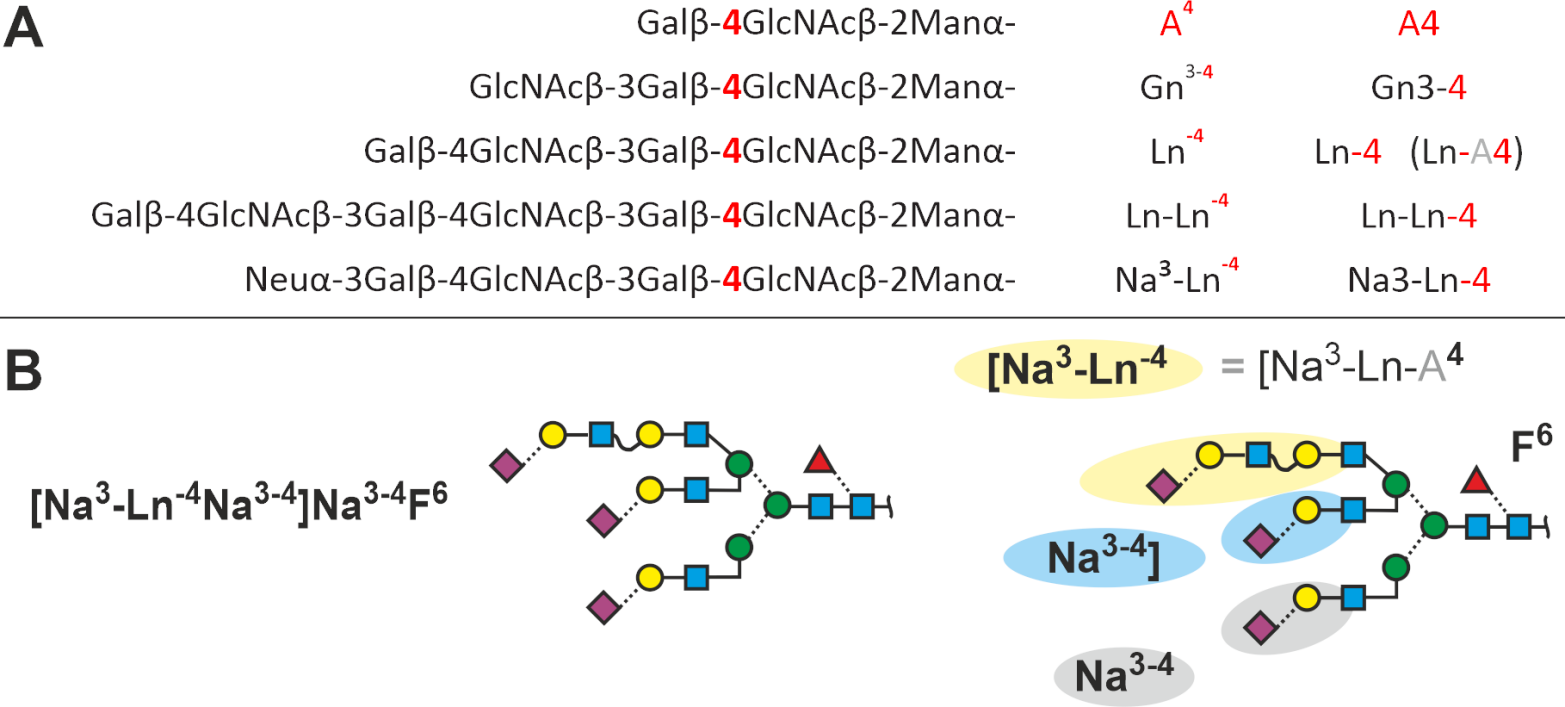
Proglycan annotation of LacNAc-repeats. A: Development of the annotation showing the acronyms both with and without superscripts. Grey parts shall remind of the “hidden galactose” residue in the term “-4”. B: Structure **N3.7.2B** found in recombinant human erythropoietin [46]. The cartoon on the right side exemplifies the 4 sub-terms of the abbreviation and, again, the “hidden galactose”.

The primary LacNAc disaccharide Galβ1-4GlcNAcβ1- that is linked to a mannose can be further elongated by the addition of GlcNAc (in β1-3 linkage to Gal), which usually is followed by the quick addition of Gal to arrive at another Galβ1-4GlcNAcβ1- (= LacNAc) unit. The following annotation scheme is a suggestion that may be substituted by a better option. Such LacNAc repeats occur in recombinant erythropoietin [46] where the root galactose was found in 4-linkage. To allow for the occurrence of 3-linked galactose, the superscript “4” is part of the term used here. The large and complex structures **N3.7.2B** in recombinant human erythropoietin [46], aka EPO will thus be written as shown in Figure 8.

### Special structures in brain and bladder

**Figure 9:**
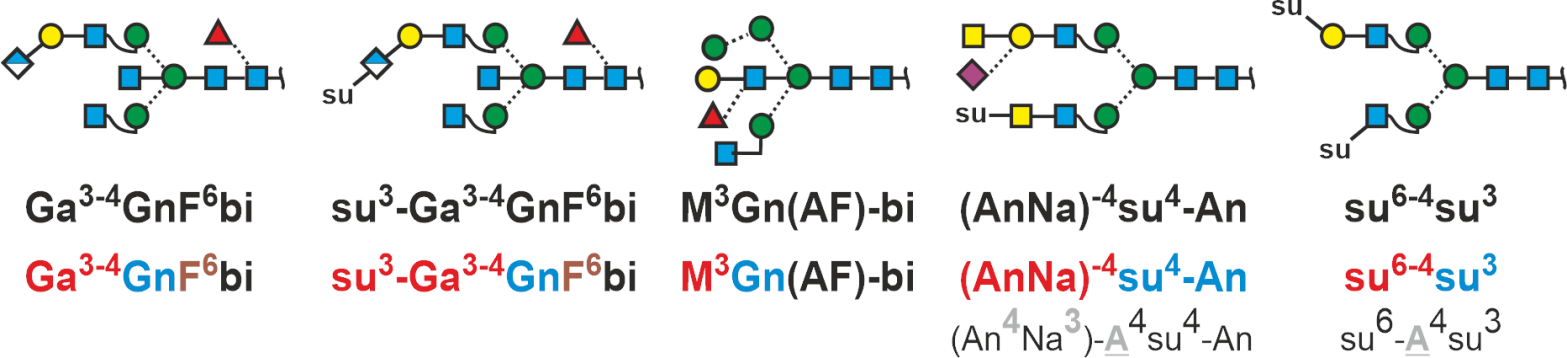
Proglycan codes for HNK-1 structures, a brain glycan with substituted bisecting GlcNAc, a sulfated SDa antigen containing glycan from uromodulin and a hypothetical sulfated N-glycan. The colored codes are just for illustration. The bottom line reveals the hidden, invariable informations.

The human brain contains sizable amounts of glycans with the “HNK-1” (from *human natural killer cells*) with sulfated glucuronic acid [47]. Annotating a structure like this requires some form of linear code and the addition of abbreviations for non-sugar substituents, in this case sulfate. Note that the hyphen binds the “su” to “Ga”, which in turn is hyphenated to the “4” (or ”3”), which stands for the regular antennary galactose (Figure 9).

Another peculiar structure is that with a Lewis X determinant in the bisecting position [26]. With the rules established so far, even such an exotic item can be named (Figure 9).

The bladder protein uromodulin aka Tamm-Horsefall protein contains glycans with sulfated GalNAc and the Sd^a^ determinant, which harbors a branch on the galactose residue [48–49]. Based on the rules for Lewis determinants, we use a round bracket embracing the two terminal residues, which do need linkage specifiers as GalNAc has only ever been found in β1-4 linkage. Neu5Ac has no other choice than the 3-position. The two residues are linked to a galactose, hence a hyphen and the pertinent linkage (Figure 9). The letter A itself is hidden as in sialylated antennae (Figure 3).

Finally, a hypothetical N-glycan shall exemplify the annotation of different sequences of sulfated antennae. To facilitate deciphering of these terms, the abbreviations are also given with colors for the 6-arm, 3-arm, and the first extension term (Figure 9).

### High-mannose and hybrid-type N-glycans

**Figure 10:**
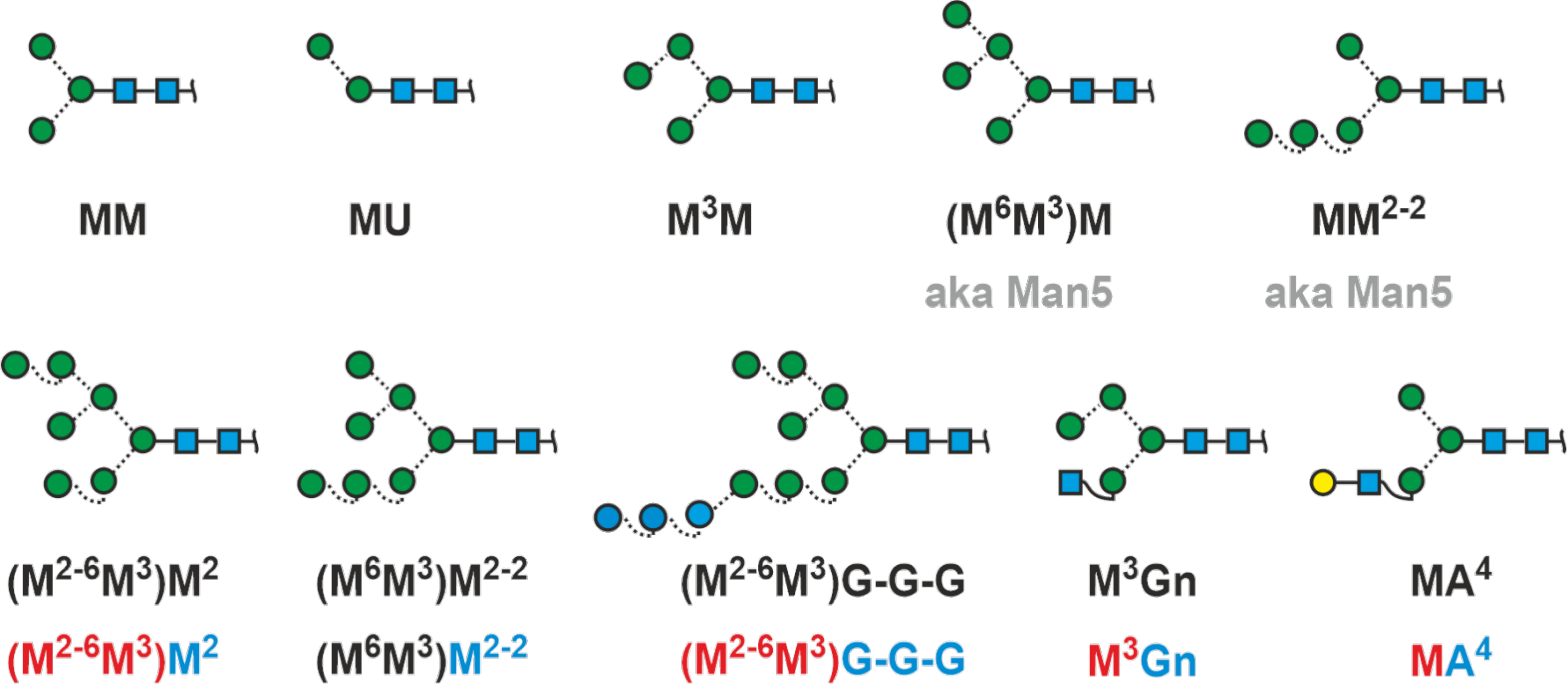
Proglycan annotation of oligomannosidic and some hybrid-type N-glycans.

The above defined rules can also be applied to high-mannose, aka oligomannosidic-type glycans, but this only makes sense if the true structure of a glycan is known, e.g., if glycans are analyzed by porous graphitic carbon chromatography [50]. Only the mannoses that occur in addition to those on the common core have to be explicitly defined and this is realized by their linkage. Noteworthy, the number of numeric figures gives the number of mannose residues in addition to the three of the core. The example (M^6^M^3^)M^2^ contains three numbers and hence describes a hexasaccharide (Figure 10).

### Plant and insect glycans

**Figure 11:**
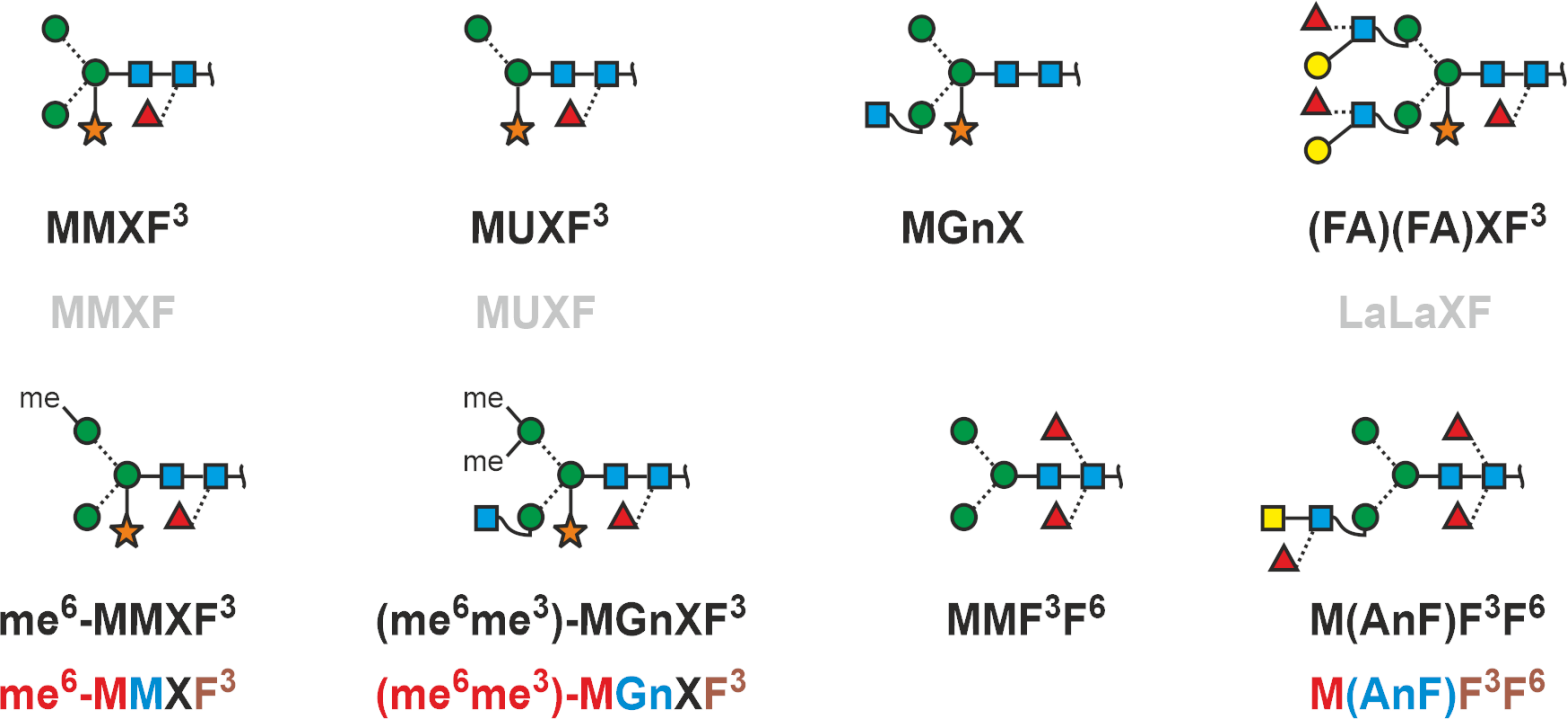
Plant and insect N‐glycans. Since the xylose strongly indicates plant N‐glycans, a simplified annotation, possible with the macro‐term “La” for the Lewis A determinant, may be used (grey font).

This chapter arises from the authors´ long involvement with glycoproteins from plants and insects, where core fucose occurs in α1,3-linkage and where – in plants – a xylose is linked to the β-mannosyl residue of the core. These two peculiarities can easily be included as shown in the examples below (Figure 11). Mosses contain structures with methyl groups [51], non-vertebrates contain numerous “unusual” and remarkable structural features such as methylation, sulfation, and zwitterionic non-sugar substituents, again glucuronic acid and often unusual architectures such as substituted core-fucose just as an example [44,52]. While the methylated moss glycans are accessible to the proglycan systems (Figure 11) the extremely diverse and unusual structures found in invertebrates or algae demand IUPAC code or graphical representations [44,53–55].

Moss N‐glycans are included here to demonstrate the ability of the proglycan system to annotate nonsugar substituents.

### Handling of ambiguities

**Scheme 1:**
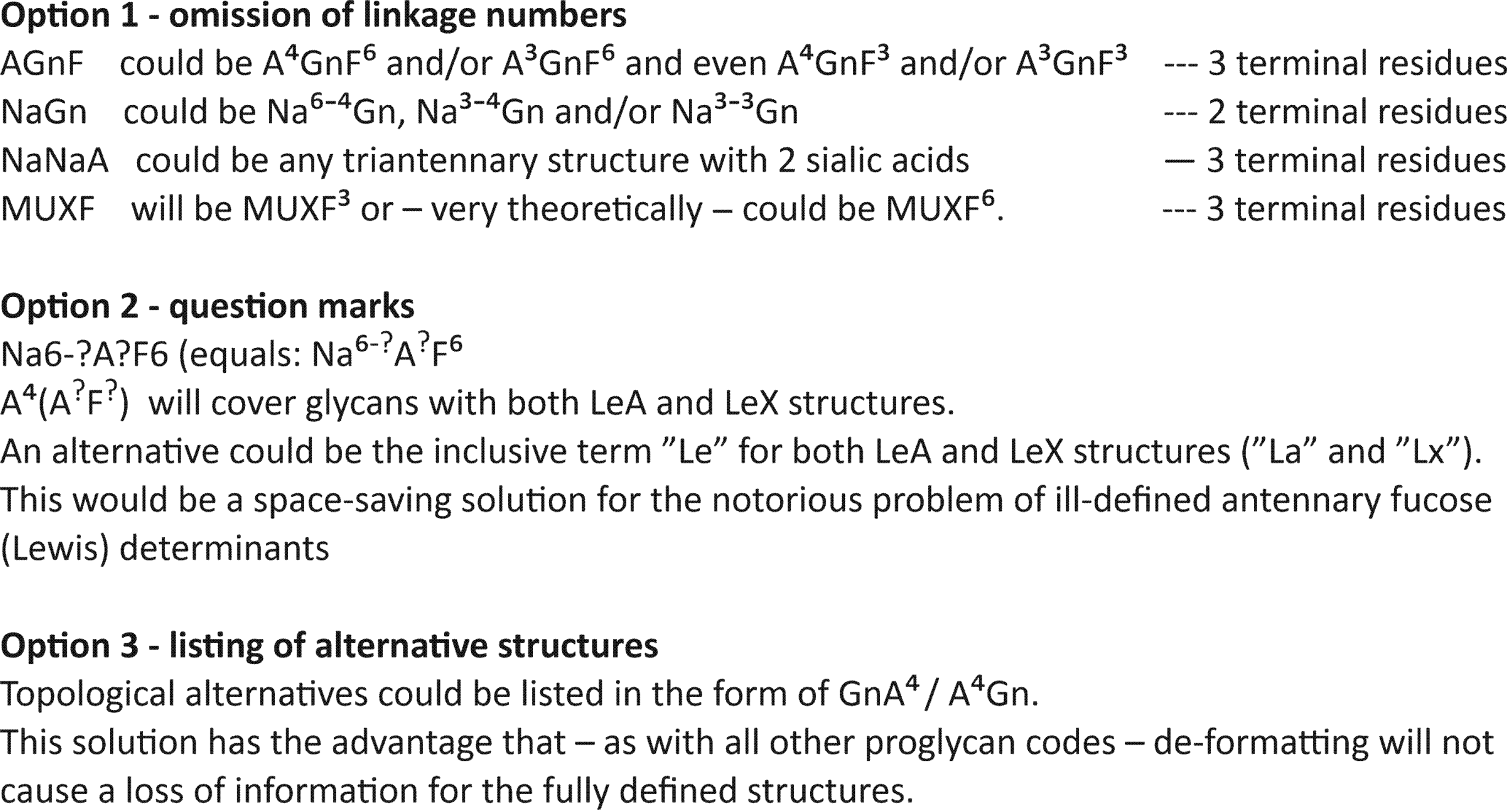
Suggestions for the depiction of incompletely defined N-glycan structures.

A disadvantage of the herein described annotation scheme is its unambiguity. A given string stands for one particular structure. All too often, groups of isomers are to be addressed or the exact structure from a group of isomers cannot be defined. This chapter refrains from defining an ultimate solution to this dilemma as we think it is not yet time to impose another layer of complexity onto the herein proposed model. On top, automatic converters will encounter difficulties with such side rules. Nevertheless, for “domestic needs” shall four options be presented (Scheme 1).

#### Attempt at an application to O-glycans

**Figure 12:**
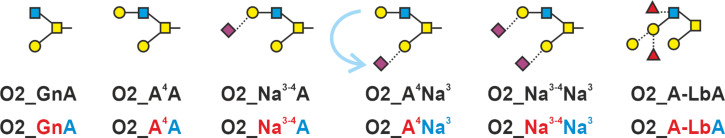
Provisional outline of how O-glycans with the frequent core 2 structure could be named with the proglycan logic.

The short, but varied core-region of O-glycans do not lend themselves to the proglycan logic as much as N-glycans. However, its application is possible as demonstrated in an article on chondrocyte glycans [56]. The branches to the core structures can be dealt with in much the same way as N-glycan antennae. It appears, however, that the particular core type has to be specified as shown in Figure 12. The very simple and frequently occurring O-glycans T-, Tn- and Sialyl-Tn-antigen may be exempted from such attempts. Likewise, will the rarer core types be left out for the time being.

### Comparison with existing abbreviation systems

Finally, we shall compare the herein portrayed naming system with existing ones. There are several systems of which a few occur more often. The most stringent and rigorous way to annotated mass spectrometry data is to give the sum formula without even trying to interpret it in terms of types of hexoses, N-acetylhexosamines (HexNAcs) etc.

Example for *m/z* = 2369.84:

Hex5HexNAc4Neu5Ac2dHex1,

or H5N4S2F1 as, e.g., in [57–59],

or 5_4_2_1 or 5421 as, e.g., in [60] and [61], respectively,

or as in web.expasy.org/glycomod/ (Hex)_2_ (HexNAc)_2_ (Deoxyhexose)_1_ (NeuAc)_2_ + (Man)_3_(GlcNAc)_2_.

These annotation types strictly avoid any overinterpretation of data and should be the explicit starting point of any other naming or drawing exercise. Unfortunately, they neither transport structural information nor do they spoil the human eye. MS spectra evaluation software thus often essentially neglects the overinterpretation problem and suggests a particular structure, where actually a range of isomers is possible as recently elaborated for the H5N4F1 composition [26]. A few more prominent alphanumerical codes were developed and shall be described and compared in the following chapter.

The “antibody glycan code” is a perfectly fine convention in the field of – surprise – antibodies, in particular recombinant IgGs, where G2F [62] stands for the therein relevant isomer A^4^A^4^F^6^ and not for any of the 40–50 possible other isobars [26].

The “Oxford code” and its variants likewise have a definite raison d'être especially when rather large and only partially defined structures shall be named, e.g., the term 3A2SF comprises a number of related structures that are most often not told apart by the analytical results [57–59].

When particular, exactly known N-glycan structures are to be described, the elaborate “Oxford code” is an option, but the proglycan system appears to be a big step ahead. The following table (Table 1) tries to give an overview of abbreviation systems. By no means does it claim to be comprehensive. The readers’ contribution to update and complete this table is highly encouraged.

**Table 1:** Examples of N-glycan abbreviation systems with assessment of the respective abilities.

	reference^a^	number of antennae	sialic acid linkage	type of sialic acid	galactose linkage	fucose linkage	arm location

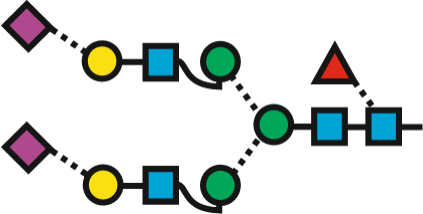							
Na^6-4^Na^6-4^F^6^	proglycan	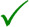	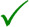	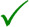	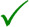	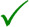	n.a.
“**antibody glycan code**” counting the number of galactose residues for biantennary glycans only							
G2FS2	[18,63–64]	( 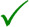 )*	x	x	x	x	n.a.
G2FS2α(2,6)	[18,65]	( 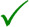 )*	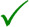	x	x	x	n.a.
“**Oxford code**” counting number of antennae, galactoses and sialic acids with **fucose first**							
FA2G2S2	[23,63]	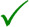	x	( 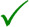 )	x	x	n.a.
F(6)A2G2S(6)2	[22]	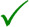	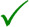	( 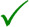 )	x	( 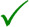 )	n.a.
“**Oxford code**” counting number of antennae, galactoses and sialic acids with **fucose last**							
A2S2F	[65]	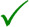	x	( 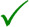 )	x	x	n.a.
A2S2G2F ^b^	[20,66]	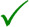	x	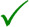	(x)	x	n.a.
A2G2FS2 ^b^	[21]	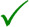	x	( 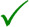 )	(x)	x	n.a.
+ bisect. GlcNAc A2G2FBS2(6)	[67]	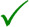	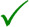	( 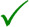 )	(x)	x	n.a.
**proglycan**							
Na^6-4^Na^6-4^F^6^	proglycan	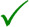	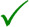	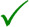	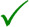	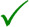	n.a.
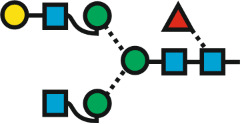							
A^4^GnF^6^	proglycan	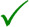	n.a.	n.a.	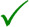	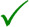	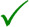
**“Elaborate Oxford code”** ^c^							
F(6)A2[6]G(4)1	[19]	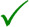	n.a.	n.a.	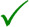	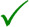	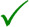

^a^References are examples without claim of completeness. ^b^Order of symbols not fixed. ^c^Somehow event-related additions are used to exactly specify structures, e.g., when α-Gal or *N*-glycolyl-neuraminic acid occur [20] or a large number of isomers are to be named [68].

### The proglycan code and the glyco-IT world

Because of its stringent logic, the proglycan codes could be generated from and converted into any other annotation format. Writing appropriate code can be a challenging task for master students. The following deliberation suggests that such an endeavor could well be of practical importance.

Developed to be a human and keyboard-friendly alternative to machine codes, the proglycan code nevertheless qualifies as a valuable component of glycan databases. A simple text search can retrieve all N-glycan structures with a particular terminal motif with a uniquely low technical threshold both for the user and the software developer. Proglycan codes inherently would be generated for fully defined structures only. Thereby, they would *en passant* act as a redundancy filter hiding the innumerable incompletely characterized entries.

## Perspective

This exposition is not intended to grimly wipe away any other type of annotation. We imagine that it can be of value wherever defined structures shall be named and written down. With increasing uncertainty, any of the systems mentioned in Table 1 may be preferred up to the point where a mere listing of the constituent monosaccharides (e.g., in the form of H5N4F2S1) might be considered as the most suitable annotation. Finally, and for the years to come, a platform for suggestions, updates and related information can be found at https://www.proglycan.info.

## Supporting Information

File 1 Possible structure terms.

## Data Availability

Data sharing is not applicable as no new data was generated or analyzed in this study.
