# Supplementary material for: Introduction of a human- and keyboard-friendly N-glycan nomenclature
Source: Beilstein J Org Chem. 2024 Mar 15;20:607–20. doi: 10.3762/bjoc.20.53 (PMC10949011; doi:10.3762/bjoc.20.53)
Supplement: File 1 — Possible structure terms. [file Beilstein_J_Org_Chem-20-607-s001.pdf]

BEILSTEIN JOURNAL OF ORGANIC CHEMISTRY

Supporting Information

for

Introduction of a human- and keyboard-friendly N-glycan nomenclature

Friedrich Altmann, Johannes Helm, Martin Pabst and Johannes Stadlmann

Beilstein J. Org. Chem. **2024,** *20,* 607–620. doi:10.3762/bjoc.20.53

Possible structure terms

License and Terms: This is a supporting information file under the terms of the Creative Commons Attribution License (https://creativecommons.org/licenses/by/4.0). Please note that the reuse, redistribution and reproduction in particular requires that the author(s) and source are credited and that individual graphics may be subject to special legal provisions.
The license is subject to the *Beilstein Journal of Organic Chemistry* terms and conditions: (https://www.beilstein-journals.org/bjoc/terms)

Appendix: A few possible "structure terms":

6-arm term

Core and bisection

3 arm term

<u>Terms possible in both first (6-arm) and second (3-arm) position:</u>

Gn	a GlcNAc linked to the invariant pentasaccharide core
U	indicates no substituent of the β-mannose.
A	Galactose linked to GlcNAc, linkage not specified
A^4 / A4	Galactose β1,4-linked to GlcNAc
A^3 / A3	Galactose β1,3-linked to GlcNAc
An^4 / An4	GalNAc β1,4-linked to GlcNAc
Na	Sialic acid linked to Gal-GlcNAc, linkages not specified
$Na^{6\text{-}4}$ / Na6-4	Neu5Ac-α2,6-Gal-1,4-GlcNAc-
$Na^{3\text{-}3}$ / Na3-3	Neu5Ac-α2,3-Gal-1,3-GlcNAc-
($Na^6Na^{3\text{-}3}$)	Neu5Ac-α2,6(Neu5Ac-α2,3-Gal-1,3-)GlcNAc- (as in fetuin)
(AF) / (A^4F^3) / (A4F3) / Lx	Lewis X determinant
(FA) / (F^4A^3) / (F4A3) / La	Lewis A determinant
$F^{2\text{-}3}$ / Lh^3	Blood group H determinant on type I chain
$F^{2\text{-}4}$ / Lh^4	Blood group H determinant on type II chain
La	Lewis A determinant = (FA)
Lb	Lewis B determinant = (FA)
Lh^3	Blood group H determinant on type I chain = $F^{2\text{-}3}$
Lh^4	Blood group H determinant on type II chain = $F^{2\text{-}4}$
Ln	LacNAc extension (Ln-, Ln^3, Ln^4)
Lx	Lewis X determinant
Ly	Lewis Y determinant
A-Lb	alpha-Galactose bound to Lewis B = *aka* B Le^b
An-Lh^4	N-acetylgalactosamine on a blood group H scaffold = Type II blood group A

<u>Possible in first (6-arm) position:</u>

M	6-arm mannose of the invariant pentasaccharide core
M^3 / M3	a mannose in 3-linkage to 6-arm mannose of the core
M^6 / M6	a mannose in 6-linkage to 6-arm mannose of the core; rare
[GnGn]	GlcNAc-β1,6-(GlcNAc-β1,2-)Man
[A^4A^4]	branched 6-arm with two Gal residues (in analogy to [GnGn])
[$Na^{6\text{-}4}Na^{6\text{-}4}$]	branched 6-arm with two terminal sialic acids (in analogy to [GnGn])
etc.	

<u>Possible in second (3-arm) position</u>:

M	3-arm mannose of the invariant pentasaccharide core

M^2 / M2	a mannose in 2-linkage to the invariant pentasaccharide core
$M^{2\text{-}2}$ / M2-2	two mannoses in series
[GnGn]	GlcNAc-β1,4-(GlcNAc-β1,2-)Man (in analogy to [GnGn])
[A^4A^4]	branched 3-arm with two Gal residues (in analogy to [GnGn])
[$Na^{6\text{-}4}Na^{6\text{-}4}$]	branched 3-arm with two terminal sialic acids (in analogy to [GnGn])
etc.	

<u>Possible in the extension positions:</u> – if present; fucosylation before bisection

F^6 / F6	α1,6-fucosylation of reducing end GlcNAc
F^3 / F3	α1,3-fucosylation of reducing end GlcNAc
F^3F^6 / F3F6	difucosylation of reducing end GlcNAc
X	β1,2-xylosylation of the β-mannose (written before fucosylation)
bi	indicates presence of a bisecting GlcNAc
A-bi	bisecting LacNAc
(AF)-bi	bisecting Lewis X

<u>Order of extension terms</u>:

[1] Xyl – [2] substituents of reducing GlcNAc (3 before 6) – [3] bisecting GlcNAc

<u>Non-sugar substituents</u>

ac	acetyl
me	methyl
pc	phosphocholine
pe	phosphoethanolamine
po	phosphate
su	sulfate
….	
